# Supplementary material for: Effects of controversial contexts on opinion changes through discussion and evaluation of decisions: A group decision experiment regarding the issue of removed soil in Fukushima
Source: PLoS One. 2026 Apr 7;21(4):e0346574. doi: 10.1371/journal.pone.0346574 (PMC13056183; doi:10.1371/journal.pone.0346574)
Supplement: S1 Appendix — (PDF) [file pone.0346574.s002.pdf]

Group discussion experiment on the final disposal of the removed soil outside of Fukushima

Faculty of Humanities and Human Sciences, Hokkaido University

Informed materials

INTRODUCTION

- Today, you are asked to discuss the "final disposal of the removed soil" and to reach a conclusion.
- The removed soil is currently being collected and managed at an interim storage facility in Okuma Town and Futaba Town, Fukushima Prefecture.
- For the next 10 minutes, we will explain the topic for our discussion.
- Following that, we will present the discussion points.
- There is no just correct conclusion, so please share your honest opinions and engage in discussion.

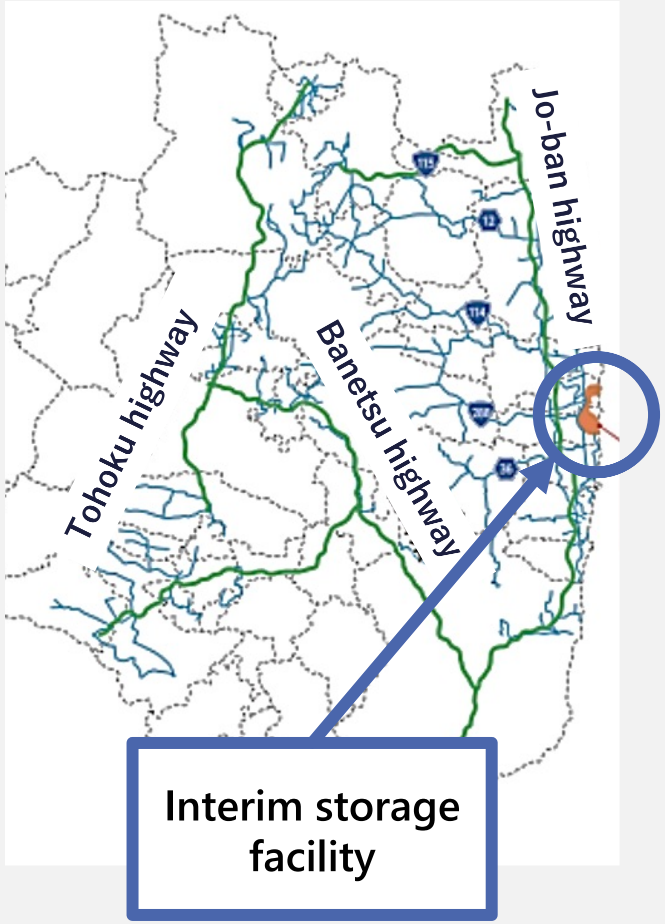

BACKGROUND

- March 2011: The accident at the Fukushima Daiichi Nuclear Power Plant generated radioactive waste and contaminated soil, primarily in Fukushima Prefecture.

  Decontamination works have been carried out across a wide area of Fukushima Prefecture, resulting in the generation of vast amounts of removed soil and other materials.

- March 2015–present: The removed soil was managed and stored at the interim storage facility in Okuma Town and Futaba Town, Fukushima Prefecture

  The final disposal of the removed soil is to be conducted outside of Fukushima Prefecture by 2045

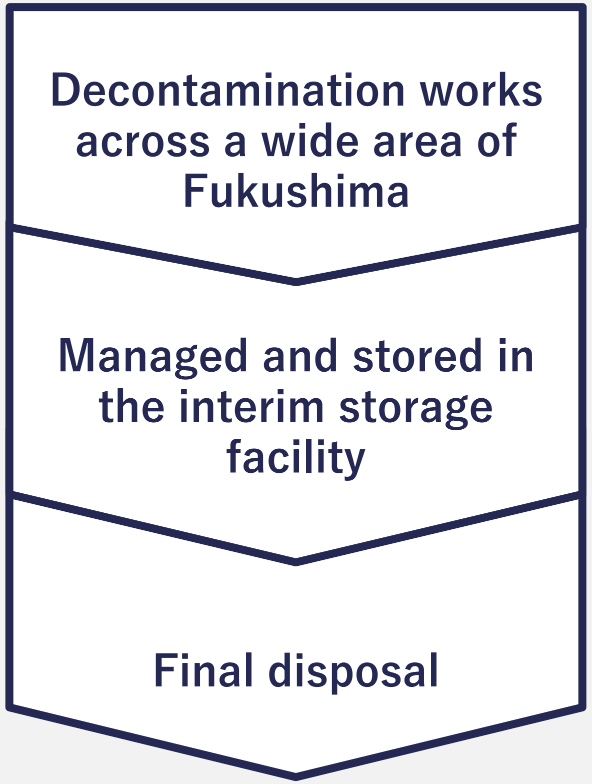

Reference: https://www.jesconet.co.jp/interim/transport/transportplan.html

FLOW FROM DECONTAMINATION WORK TO FINAL DISPOSAL

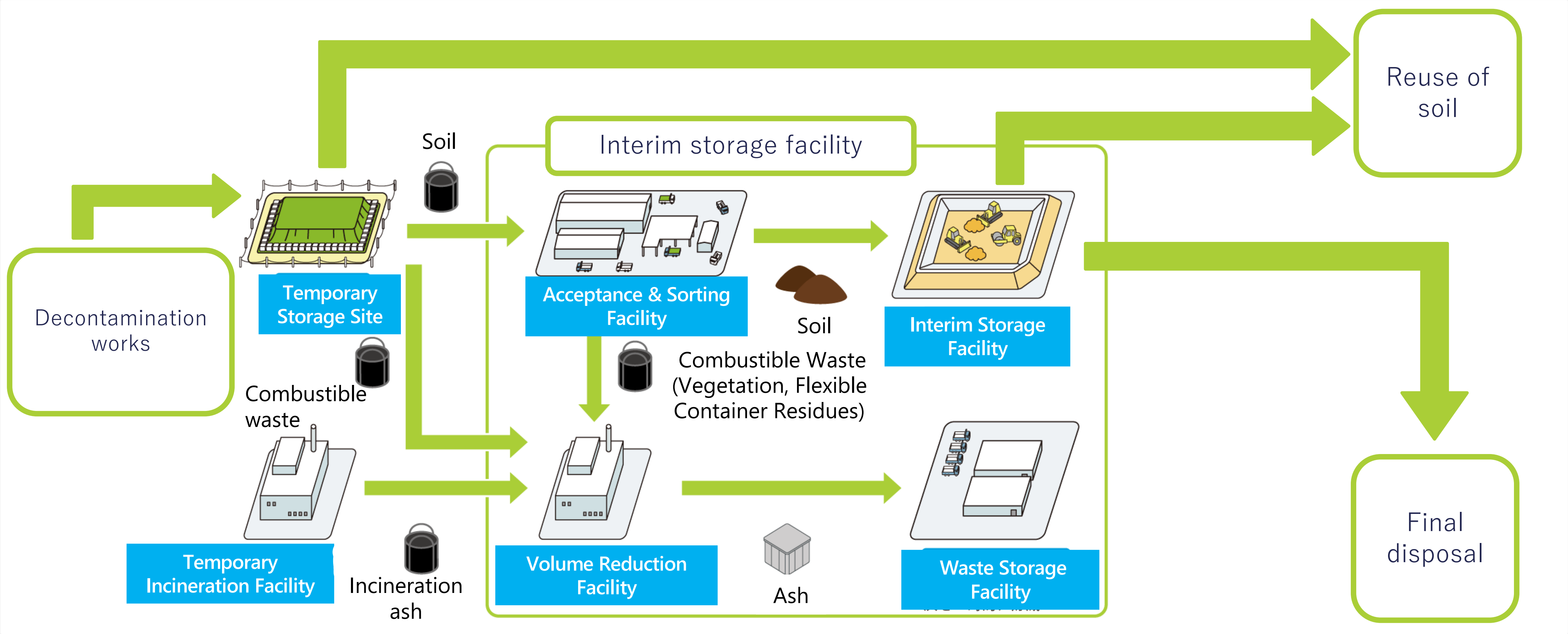

Reference: http://josen.env.go.jp/chukanchozou/about/ (Added arrows and text)

DECONTAMINATION WORK FOLLOWING THE NUCLEAR ACCIDENT

- Following the nuclear accident, decontamination efforts were conducted over a wide area of Fukushima Prefecture.

  The decontamination works have generated vast amounts of removed soil and other materials.

- A facility is needed to safely and centrally manage and store them until their final disposal.

  An interim storage facility was constructed in Okuma Town and Futaba Town, Fukushima Prefecture.

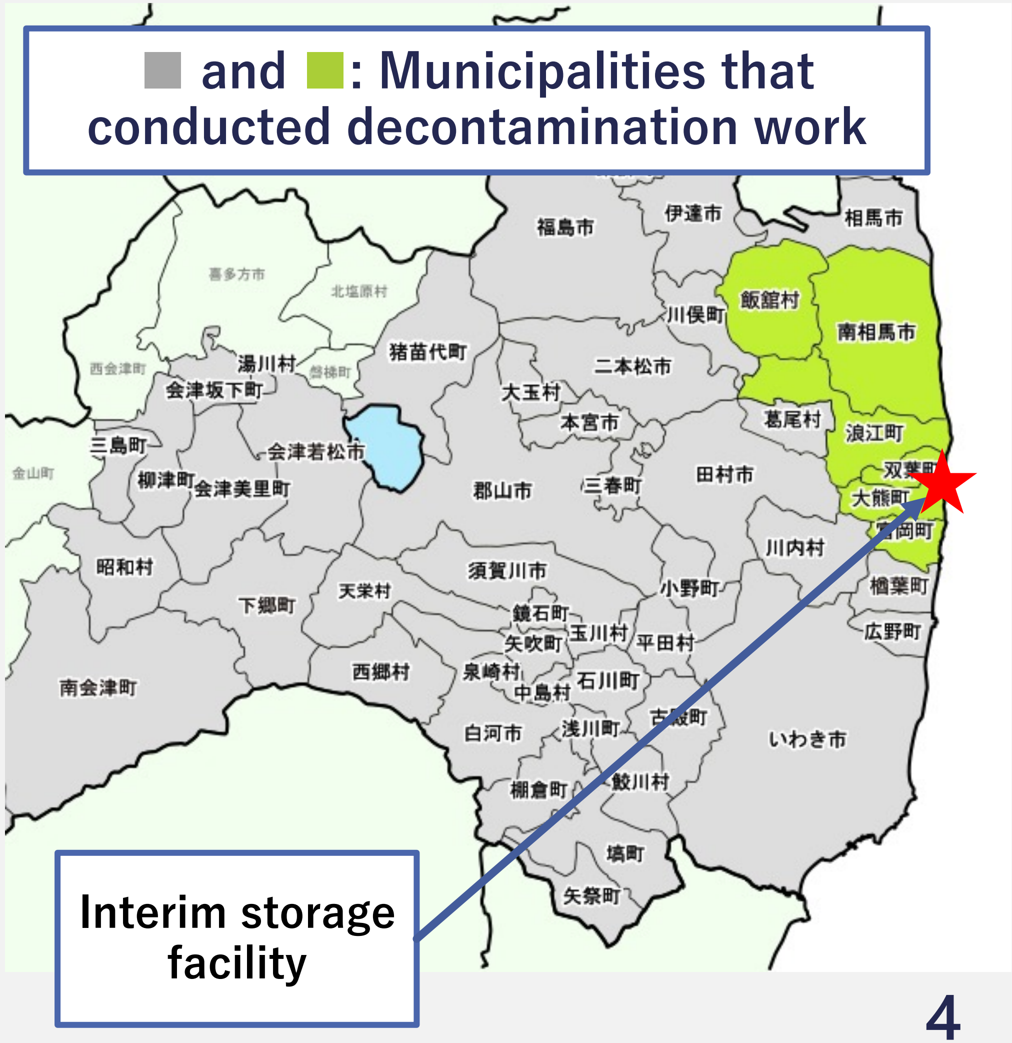

Reference: http://josen.env.go.jp/chukanchozou/transportation/index.html

OVERVIEW OF THE INTERIM STORAGE FACILITY

- Location: Okuma Town and Futaba Town, Fukushima Prefecture
- Site area: Approximately 16 km^2

  About the same size as Shibuya Ward, Tokyo, Japan

  About 80% is privately owned land
- Before the accident, there were rice paddies and a shrine.

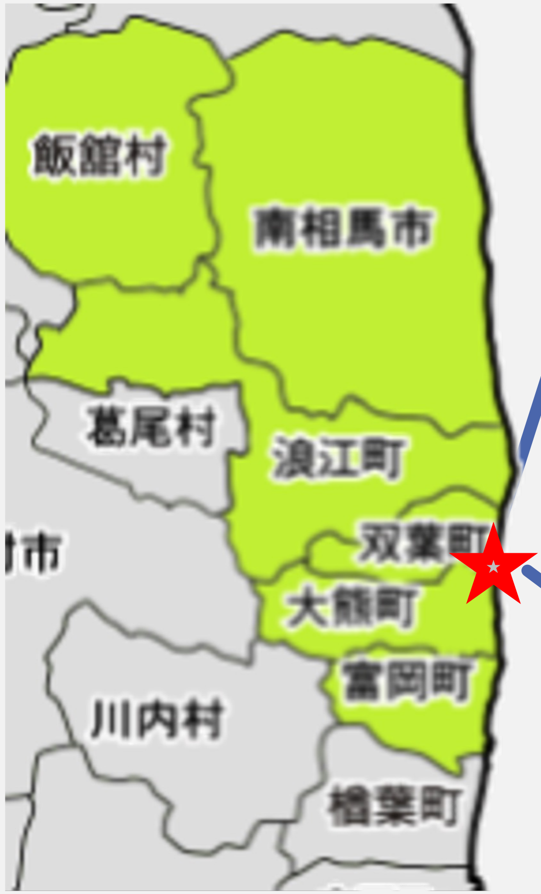

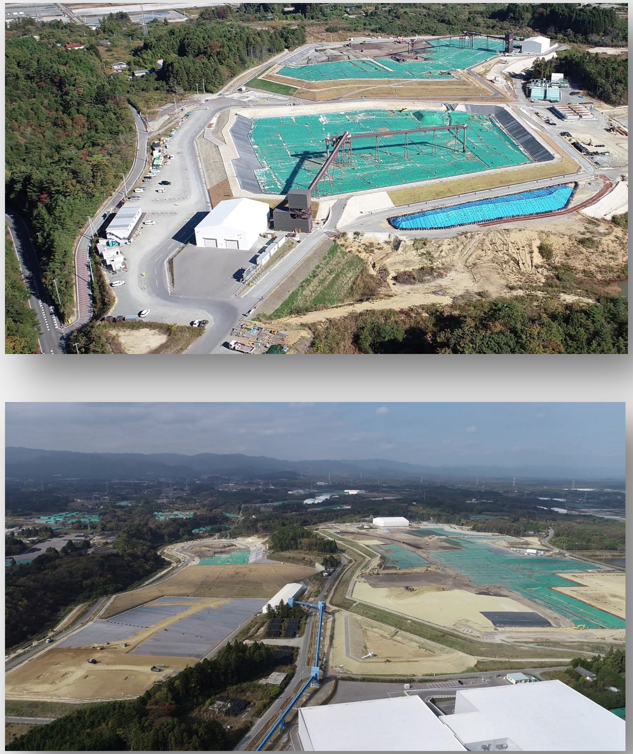

Interim Storage Facility (Top: Futaba Town, Bottom: Okuma Town)

Reference:
http://josen.env.go.jp/chukanchozou/transportation/index.html
http://josen.env.go.jp/chukanchozou/about/
https://www.jesconet.co.jp/interim_infocenter/

ABOUT OKUMA TOWN AND FUTABA TOWN, FUKUSHIMA PREFECTURE

- Population of Okuma and Futaba towns

	At the time of the earthquake	Number of evacuants (as of December 1, 2022)
Okuma town	11,505 people	9,616 people
Futaba town	6,939 people	6,639 people

- In 2017, “Designated Reconstruction and Revitalization Base Areas” were established in Okuma Town and Futaba Town, within the areas where evacuation orders had been lifted, allowing residents to return and live there.

Evacuation orders for the “Designated Reconstruction and Revitalization Base Areas” had been lifted in Okuma Town in June 2022 and in Futaba Town in August of the same year.

However, some areas within both towns remain designated as areas where return is difficult.

Reference:
https://www.town.okuma.fukushima.jp/soshiki/jumin/22955.html
https://www.town.fukushima-futaba.lg.jp/10291.htm

STATEMENTS BY THE MAYORS OF OKUMA TOWN AND FUTABA TOWN

FROM THE DIALOGUE FORUM ORGANIZED BY THE MINISTRY OF ENVIRONMENT (MAY 2021)

- When the idea of hosting the interim storage facility first arose, every resident had their own feelings about our hometown—some wanted to protect the land passed down from their ancestors, while others felt that returning was simply impossible.
- Accepting the facility meant, on top of the long evacuation we'd already endured since the accident, having to give up our inherited land, homes, and property.
- I'll never forget the anger, sadness, and pain I saw on the faces of our residents at that time.
- In the end, we knew that for Fukushima's recovery, someone had to take on the burden of storing this removed soil, and with that painful conviction, we agreed to host the facility.
- Every landowner had their own struggles and emotions.
- And yet, so many people still chose to cooperate. Because of that, we've been able to make progress in restoring the environment here in Fukushima, which was so deeply damaged by the nuclear accident.

QUANTITY AND RADIOACTIVE CONCENTRATION OF THE REMOVED SOIL

- The volume of removed soil and other materials delivered to the interim storage facility was approximately 13.38 million cubic meters (as of December 2022)
- Approximately 25% of the removed soil had radioactive cesium concentrations exceeding 8,000 Bq/kg, while the remaining 75% had concentrations at or below this level.

  8,000 Bq/kg is a level that does not exceed the international safety standard (1 mSv/year) when assessing radiation exposure across all processes up to final disposal, when evaluating radiation exposure safety.

※Bq: The higher the value, the more radiation is emitted from that point.

※Sv: A unit of measurement for radiation exposure to the human body. The higher the value, the greater the impact of radiation on the human body.

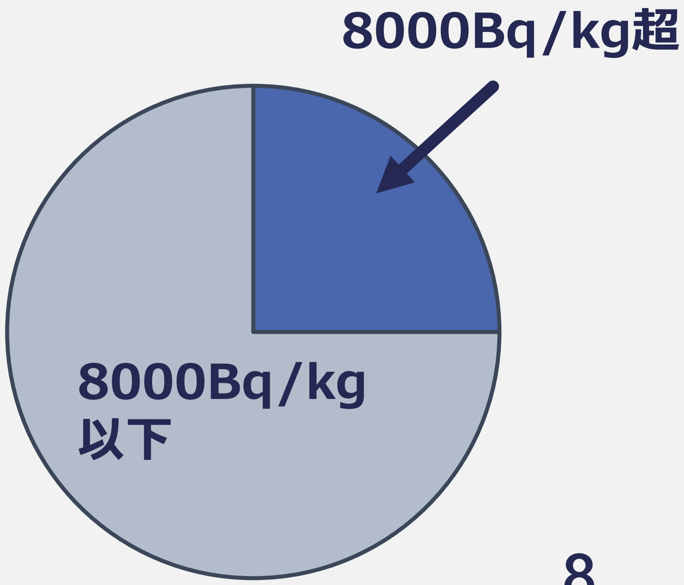

Reference: http://josen.env.go.jp/chukanchozou/material/pdf/removed-soil_recycling_2205.pdf

CLASSIFICATION OF REMOVED SOIL

Removed soil
below 8000 Bq/kg

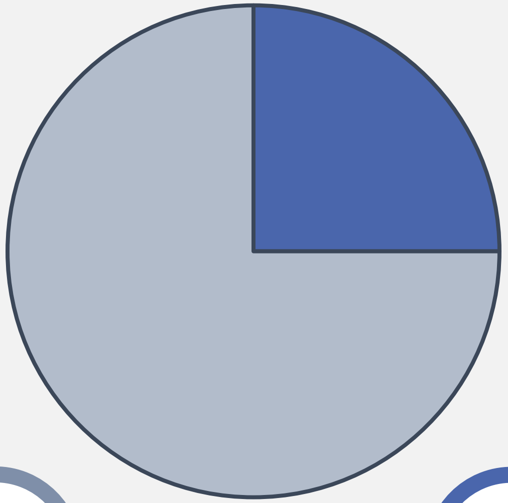

Removed soil
exceeding 8,000 Bq/kg

Reuse

Public works projects and reuse on farmland

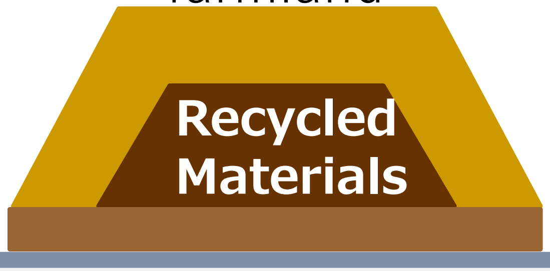

Final disposal

Completion of the final disposal outside of Fukushima within 30 years after the start of management in the interim storage facility (by 2045)

Reference: http://shiteihaiki.env.go.jp/fukushimamirai/sonosaki/dialogue/report_210911/pdf/report01_01.pdf

THE RELATIONSHIP BETWEEN REUSE AND FINAL DISPOSAL

- Recycling reduces the amount of soil requiring final disposal.

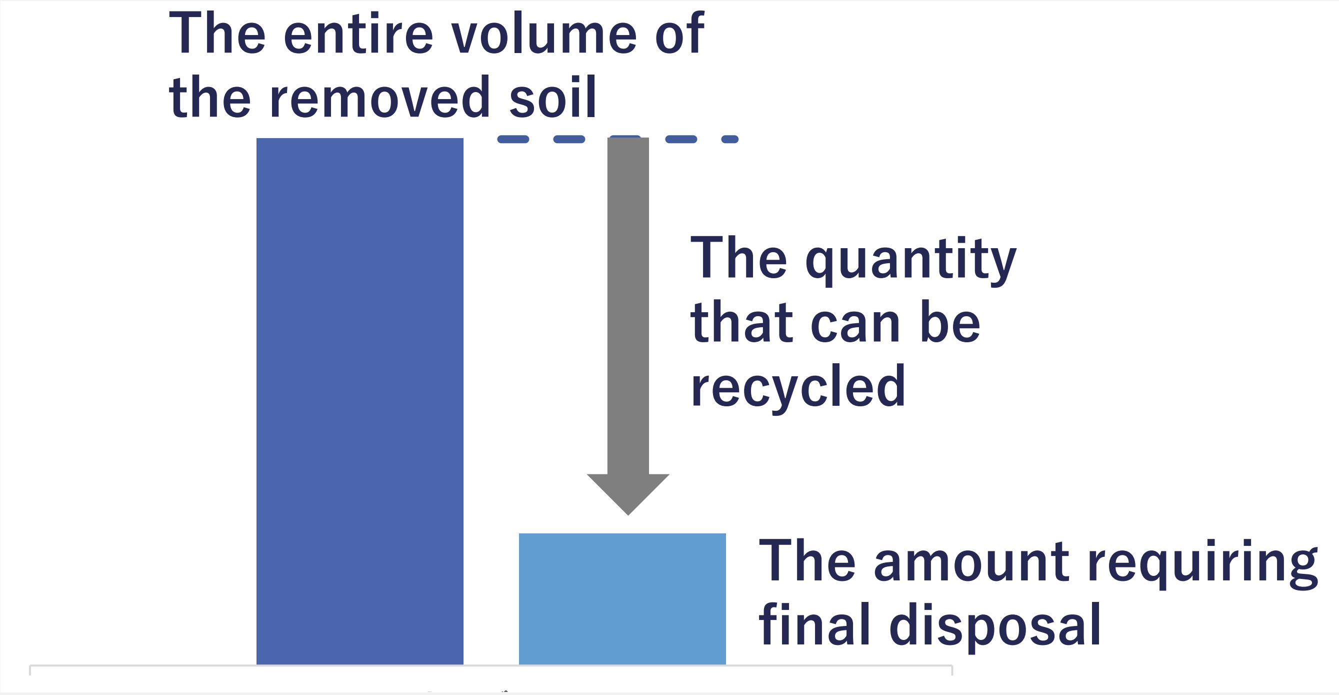

THE GOVERNMENT'S BASIC POLICY FOR FINAL DISPOSAL

- The government's responsibility to complete the final disposal of the removed soil outside of Fukushima by 2045 is stipulated by law.
- To facilitate the realization of final disposal outside of Fukushima, it is necessary to reduce the amount of waste requiring final disposal and scale down the size of the facilities.

  The national policy is to reuse low-level radioactive soil for recycling as construction materials, provided safety is ensured, to reduce the volume of waste requiring final disposal.
- **Regarding the final disposal, specific methods and disposal candidate sites have not yet been determined.**

Reference: http://josen.env.go.jp/chukanchozou/facility/effort/

CRITICISM OF THE LAW

- It is unclear how the law (regarding the final disposal of the removed soil outside of Fukushima by 2045) was enacted

  The Cabinet decided that "as a result of comprehensive judgment, final disposal will be conducted outside of Fukushima Prefecture."

- Regarding reuse of the removed soil, the legal basis is ambiguous.

  "Recycling" constitutes disposal (though not "final disposal").

DISCUSSION POINTS RELATED TO REMOVED SOIL ISSUES

- Two points to consider when discussing the disposal of removed soil

Whether to recycle

When recycling

- Number of recycling sites
- The range of the area for conducting the recycle

Number of the final disposal sites

POINT 1: RECYCLING OF THE SOIL

- The differences and similarities between recycling and final disposal are as follows:

	Feature	Concentration of removed soil	Management	Transportation cost
Recycling	Can be effectively used as materials and resources	Below 8000Bq/kg	Relatively easy	unchanged (the further away from Fukushima the higher the level)
Final disposal	There is no other option than to bury it	Over 8000Bq/kg	Strict management for a long time	

POINT 1: RECYCLING OF THE SOIL

- Recycling applications: fill materials for roads, embankments, and farmlands

  Demonstration projects have already been implemented in multiple municipalities in Fukushima Prefecture.

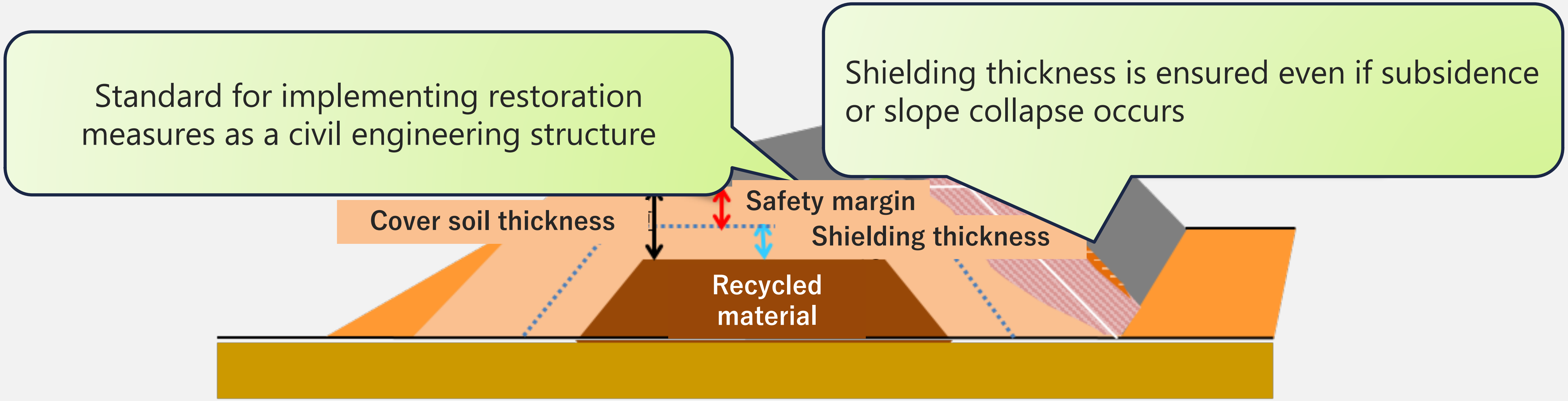

The cover soil thickness is designed to ensure adequate shielding thickness to limit radiation exposure, even when normal repairs as a civil engineering structure are carried out.

Reference: http://josen.env.go.jp/chukanchozou/facility/recycling/

EXAMPLES OF DEMONSTRATION PROJECTS

- Safety has been confirmed for all projects.

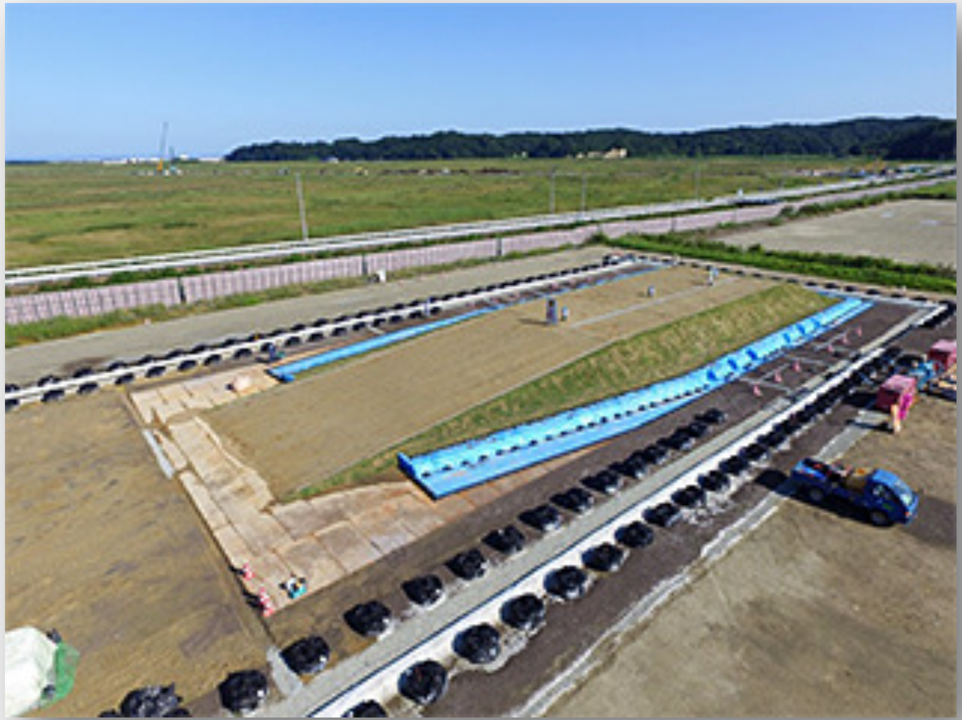

Recycling Demonstration Project at the Temporary Storage Site in Minami-soma City, Fukushima Prefecture

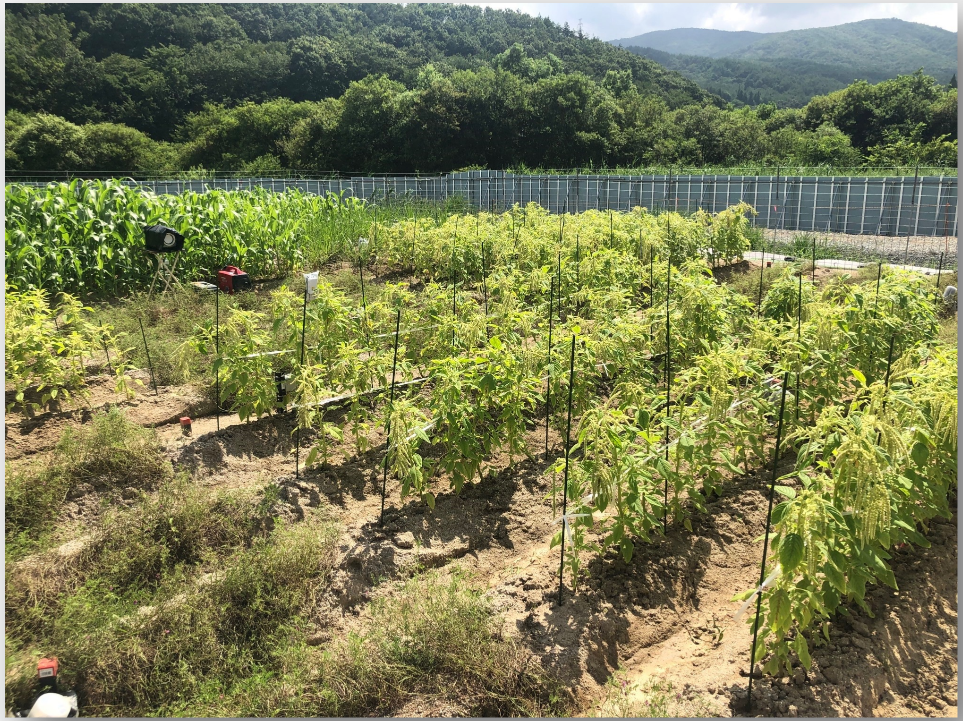

Recycling Demonstration Project in Iitate Village, Fukushima Prefecture

Reference: http://josen.env.go.jp/chukanchozou/facility/recycling/project_minamisoma/
http://josen.env.go.jp/chukanchozou/facility/recycling/project_iitate/

WHERE AND IN WHICH AREAS SHOULD RECYCLING BE IMPLEMENTED?

- How many locations will recycling be implemented at?

  It is possible to perform the procedure at one location only or at multiple locations.

	Burdens	Management	Stakeholders and residents
One location	The removed soil accumulates in specific areas, placing significant physical and psychological burdens on receiving regions.	Concentrate in one place	Relatively few
Multiple location	The removed soil is separated, reducing the physical and psychological burden on receiving areas.	Management is required at multiple locations.	Relatively more

- In which areas should recycling be implemented?

  In principle, it is possible nationwide in Japan.

  Recycling ≠ final disposal, so it is possible in Fukushima Prefecture as well.

POINT 2: NUMBER OF FINAL DISPOSAL SITES

- The number of final disposal sites has not been determined.
  Similar to recycling, but final disposal involves additional risk factors.

	Burdens	Management	Stakeholders and residents	Risk of final disposal
One location	The removed soil accumulates in specific areas, placing significant physical and psychological burdens on receiving regions.	Concentrate in one place	Relatively few	The risk of the removed soil is concentrated in one location.
Multiple location	The removed soil is separated, reducing the physical and psychological burden on receiving areas.	Management is required at multiple locations.	Relatively more	The risk of contaminated soil is distributed across multiple locations.
