# Supplementary material for: Effects of controversial contexts on opinion changes through discussion and evaluation of decisions: A group decision experiment regarding the issue of removed soil in Fukushima
Source: PLoS One. 2026 Apr 7;21(4):e0346574. doi: 10.1371/journal.pone.0346574 (PMC13056183; doi:10.1371/journal.pone.0346574)
Supplement: Attachment — Submitted filename: PLOS Review PONE-D-25-63386 2025Feb2.docx [file pone.0346574.s006.docx]

Title: **Effect of controversial contexts on opinion changes through discussion and evaluation of decisions: A group decision experiment regarding the issue of removed soil in Fukushima.**

Manuscript number: PONE-D-25-63386

General

- Biases and sources of error are only partially acknowledged in the manuscript
  - Paper would benefit from discussion of biases and how this can impact opinions
  - Motivated reasoning is a subconscious behavior that can lead to confirmation and observer / research biases – need to acknowledge and discuss these sources of error and their potential impact on study results
  - Participants were 128 Japanese university student – small demographic
  - How was survey advertised? Across campus or only in select classes? Noted a bias towards science engineering students
  - Participants were largely first-year students (>72%), so biased representation of student body at university
  - Emotions will often impact opinions, especially in competitive scenario, but in study design opinions would have only been driven by factual opinions formed from reading informational materials
  - Limited participant knowledge going in and to draw from to form opinions, so inherently difficult for them to form firm views
- Need to add section to fully address and discuss sources of uncertainties and other factors that can influence context of controversy
- Terminology
  - Consider use of “debate” as opposed to “controversy” throughout manuscript
  - Consider use of “structured” as opposed to “constructive” throughout manuscript
  - Consider use of “differences” as opposed to “conflicts” throughout manuscript
- Consider adding text highlighting importance of early and ongoing communication and engagement during projects (i.e., removed soil) to create robust deliberative processes and strengthen decision making
- Issue is disposal, but reuse option(s) were not fully discussed or considered in paper
  - Resulted in collaborative group choosing “Other”, but if study design had more fully considered reuse, strength in collaborative approach should be more apparent
  - Inability to discuss reuse options as part of study design can impact opinions and results, as it can reduce likelihood of observing transitions from competitive to collaborative participant behavior
    - Statement on Page 27 “…some groups concluded that the burden of soil disposal should be shared within the entire Tohoku region, or that disposal should take place in both uninhabited areas outside and within Fukushima Prefecture” is indicative of value added if reuse options had been more fully integrated in study design
  - Inclusion of reuse options beyond “other” in study design would have provided valuable insight into consensus building and critical thinking amongst participants in collaborative group to inform decision-making
    - Competitive condition doesn’t lend itself to consensus building
    - Pre-disposition of competitive to not accept or move toward cooperative
- Results of study should be more fully supported through comparisons to other similar studies and/or case studies –
  - There are a few references provided to case studies and laboratory experiments, but no fulsome or supportive discussion to accompany references
- Consider referring to relevant content in following Open Access publication:
  - “Democratizing Risk Governance: Bridging Science, Expertise, Deliberation and Public Values”, 2023, Monica Gattinger (editor), Palgrave Macmillan (publisher)

Specific

- Page 4: States “When the conditions for deliberation are properly established, group opinions tend to move in a moderate direction rather than become more extreme…”
  - Need to establish common ground and clear goal(s) prior to initiation of discussion
  - Balanced, but also unbiased factual information
  - Deliberations should follow a robust and structured framework / process
- Page 6: States “….when participants deliberate on diverse perspectives of issues,…”
  - Diverse perspectives mitigate biases and adds strength to decision making
- Page 8: States “…participants pursue mutually incompatible goals…”
  - Should add an example to clarify instances where goals may be incompatible
  - Sometimes more beneficial for participants to take opposing views, as controversy can often lead to additional funding and strengthen visibility vs a supportive view
- Page 8: States “...participants work together to achieve shared objectives…”
  - Situations where there is a mutual benefit to participants provides motivation to work together to achieve shared objectives – add clarification
- Research Aim and Hypotheses Section – Consider removing this section and modifying text on Page 12 where study hypotheses are shown to remove redundancy
- Page 14 Experimental Overview: States “…asked to consider both positions regardless of their initial opinion.”
  - Possible that their own biases could influence their objectivity – source of uncertainty in study design
- Table 1 and 3: Need to add more description and clarity to these tables – reader should be able to quickly view and understand results in a table without needing to delve too much into descriptive text before or after the table
- Table 4: Need to mention scale in header and link footnote (e.g., add * next to Table title and in front of foot note, place footnote directly under table). Footnote should also read “Informational materials are provided….”
- Manipulation Section would have benefited from a table to summarize approach with a short description vs over two pages of text
- Page 33: States “…it is impossible to involve such stakeholders because the regions bearing the burden of the removed soil have not yet been determined…”
  - Study design could have included scenario where participants are asked to pretend soil will be placed in the regions where they live – consider adding as future recommendation in conclusions
- Page 35: States “… cooperative controversy contexts can yield compromised solutions that reflect the perspectives of stakeholders on both sides…”
  - A solution oriented approach able to evaluate broader range of possible options and outcomes is not necessarily a compromise
