# Supplementary material for: Effects of controversial contexts on opinion changes through discussion and evaluation of decisions: A group decision experiment regarding the issue of removed soil in Fukushima
Source: PLoS One. 2026 Apr 7;21(4):e0346574. doi: 10.1371/journal.pone.0346574 (PMC13056183; doi:10.1371/journal.pone.0346574)
Supplement: Attachment — Submitted filename: Response_to_Reviewers.docx [file pone.0346574.s008.docx]

Yume Souma

Hokkaido University

North 10 west 7, Kita-Ku, Sapporo 0600810, Japan

+81 01-706-3060

mail: souma.yume.l6@elms.hokudai.ac.jp

March 16, 2026

Dear Editor and Reviewers,

Thank you very much for your thoughtful review and constructive comments on our manuscript entitled “Effects of controversial contexts on opinion changes through discussion and evaluation of decisions: A group decision experiment regarding the issue of removed soil in Fukushima” (Manuscript ID: [PONE-D-25-63386]).

We have carefully considered the reviewers' feedback and revised the manuscript accordingly.

Below, we provide a point-by-point response to each reviewer's comment.

**Response to the Review Comments in the decision letter**

- The discussion is focused on final disposal while at the beginning the issue of reuse of soil is also mentioned. It should be useful to explain the focus of the study with regard to the broader issue.
- Reply: As you correctly pointed out, our previous manuscript lacked a sufficient explanation as to why we focused on the final disposal of the removed soil. Hence, we have explained the relationship between soil reuse and final disposal in detail in the Introduction section, along with providing our rationale for focusing on final disposal (p. 12, l. 273 - p. 13, l. 282).
- As mentioned at the end in terms of limitation, the participants were “only” students. Although this is not representative of the citizens concerned with this issue, it could be interesting to highlight the usefulness to engage young generation in such a topic which has long-term challenges. In addition, the issue of solidarity between regions and accross generations could be discussed or mentioned.
- Reply: We agree that the involvement of younger generations is crucial for long-term issues, such as the management of removed soil. Therefore, we have added a discussion on this point, as well as on the importance of considering interregional and intergenerational differences, to the Discussion section (p. 37, l. 756 - p. 38, l. 762).
- Fukushima is used sometimes in reference to the Prefecture, sometimes to the NPP, sometimes it is mentioned Fukuhsima Prefecture. An homogenisation of the term used would be helpful to understand where we are.
- Reply: To avoid any confusion, we have carefully reviewed and revised our use of the term “Fukushima” throughout the manuscript. Particularly, we specified “Fukushima Prefecture” when necessary.
- On line 248, please change “limit” by “value”. This is not a dose limit, but a radiological criteria.
- Reply: We have revised the text as suggested (p. 11, l. 252).
- The length of the paper was much longer than what should be required to concisel results of their study.
- Reply: Based on your suggestion, we have made every effort to shorten the manuscript. However, as we also incorporated extensive revisions to address the reviewers’ insightful and constructive comments, the overall length could not be significantly reduced. We would appreciate your understanding in this matter.
- Study design could also have been strengthened, assumptions and limitations more fully explored, and study would have benefited from some cross comparison to case studies that used competitive and collaborative approaches to decision-making, and/or a more fulsome discussion of how results compare to similar studies or approaches conducted by other scientists.
- Reply: According to the suggestion, we strengthened the study design, assumptions and limitations more fully explored. In the Discussion section, we have added a comparison with a case study of an actual citizen participation workshop on the removed soil. The details of our revisions are provided in the following sections.

**Responses to specific comments on the manuscript**

**General**

- Biases and sources of error are only partially acknowledged in the manuscript
  - Paper would benefit from discussion of biases and how this can impact opinions
  - Motivated reasoning is a subconscious behavior that can lead to confirmation and observer / research biases – need to acknowledge and discuss these sources of error and their potential impact on study results
- Reply: We have interpreted the results regarding potential bias within the framework of motivated reasoning and added this to the Discussion section (p. 34, l. 665-680).
  - Participants were 128 Japanese university student – small demographic
  - How was survey advertised? Across campus or only in select classes? Noted a bias towards science engineering students
  - Participants were largely first-year students (>72%), so biased representation of student body at university
- Reply: We have described the participant recruitment process in the Methods section (p. 15, l. 339-341). While we acknowledge the reviewer’s concern about potential bias in participant demographics, the demographic composition was consistent across all conditions. Therefore, we believe that this did not affect the comparisons between conditions. We have also addressed this point in the Methods section (p. 16, l. 360-367).
  - Emotions will often impact opinions, especially in competitive scenario, but in study design opinions would have only been driven by factual opinions formed from reading informational materials
- Reply: While participants likely formed their opinions based on the informational materials provided, we recognize that emotional factors may also have been influential. We have discussed this possibility in the Discussion section (p. 36, l. 727-731).
  - Limited participant knowledge going in and to draw from to form opinions, so inherently difficult for them to form firm views
- Reply: In accordance with your comment, we have added a statement in the Limitations sub-section regarding the possibility that participants may have found it difficult to maintain firm or established opinions (p. 36, l. 731-p. 37, l. 734).
- Need to add section to fully address and discuss sources of uncertainties and other factors that can influence context of controversy
- Reply: We have discussed other factors that may influence opinion change and the context of controversy, which were not directly addressed in this experiment. However, owing to space constraints, we incorporated these points into the appropriate existing sections rather than adding a new section (p. 34, l. 665-680; p. 36, l. 727-731).
- Terminology
  - Consider use of “debate” as opposed to “controversy” throughout manuscript
- Reply: Thank you for your suggestion. We have revised the terminology as suggested, except in instances where we adhered to the term “constructive controversy” to maintain consistency with previous literature, as written in p. 5, l. 105. Hence, we use the term when we mean the original nuance.
  - Consider use of “structured” as opposed to “constructive” throughout manuscript
- Reply: Thank you for your suggestion. As mentioned above, we have updated the terminology in accordance with your suggestion while retaining the established term from previous literature where necessary, as written in p. 5, l. 105. Hence, we use the term when we mean the original nuance.
  - Consider use of “differences” as opposed to “conflicts” throughout manuscript
- Reply: Thank you for the suggestion. To describe the presence of diverse opinions, we have replaced the term “conflicts” with “differences” or similar where appropriate. However, we have retained the term in contexts where participants (or individuals) were irreconcilable during the discussion.
- Consider adding text highlighting importance of early and ongoing communication and engagement during projects (i.e., removed soil) to create robust deliberative processes and strengthen decision making
- Reply: Following your suggestion, we have added a statement to the Introduction section emphasizing the importance of early and ongoing communication and engagement regarding the issues of removed soil (p. 12, l. 269-272).
- Issue is disposal, but reuse option(s) were not fully discussed or considered in paper
  - Resulted in collaborative group choosing “Other”, but if study design had more fully considered reuse, strength in collaborative approach should be more apparent
  - Inability to discuss reuse options as part of study design can impact opinions and results, as it can reduce likelihood of observing transitions from competitive to collaborative participant behavior
    - Statement on Page 27 “…some groups concluded that the burden of soil disposal should be shared within the entire Tohoku region, or that disposal should take place in both uninhabited areas outside and within Fukushima Prefecture” is indicative of value added if reuse options had been more fully integrated in study design
  - Inclusion of reuse options beyond “other” in study design would have provided valuable insight into consensus building and critical thinking amongst participants in collaborative group to inform decision-making
    - Competitive condition doesn’t lend itself to consensus building
    - Pre-disposition of competitive to not accept or move toward cooperative
- Reply: As you pointed out, the previous version of the manuscript focused on whether the final disposal would be conducted outside Fukushima Prefecture and lacked sufficient explanation and discussion regarding soil reuse.
  The information materials provided to the participants included information about soil reuse. Although they were not asked to decide on soil reuse, this was explicitly presented as a relevant point for discussing final disposal. We have added these points to the Methods section (p. 19, l. 407-410; p. 21, l. 444-446).
  Additional analysis revealed that participants in both conditions evaluated soil reuse positively before the discussion and became even more positive afterward. Furthermore, text analysis of group presentations on their decisions showed that several groups reached consensus on implementing soil reuse, regardless of the conditions or group decisions on final disposal. We believe that discussing both final disposal and reuse of the removed soil prevented the escalation of conflict of opinion. We have also added these results and interpretations to the Discussion section (p. 35, l. 695 - p. 36, l. 715).
- Results of study should be more fully supported through comparisons to other similar studies and/or case studies –
  - There are a few references provided to case studies and laboratory experiments, but no fulsome or supportive discussion to accompany references
- Reply: We have cited previous laboratory experiments and case studies on constructive controversy or the removed soil issue, and we have also compared our findings with them in the Discussion section (p. 34, l. 662-663, p. 35, l. 699, p. 39, l. 781-783).
- Consider referring to relevant content in following Open Access publication:
  - “Democratizing Risk Governance: Bridging Science, Expertise, Deliberation and Public Values”, 2023, Monica Gattinger (editor), Palgrave Macmillan (publisher)
- Reply: We are grateful for this suggestion. We have now incorporated this reference into our manuscript.

**Specific**

- Page 4: States “When the conditions for deliberation are properly established, group opinions tend to move in a moderate direction rather than become more extreme…”
  - Need to establish common ground and clear goal(s) prior to initiation of discussion
  - Balanced, but also unbiased factual information
  - Deliberations should follow a robust and structured framework / process
- Reply: We understood the comment as a request for further clarification regarding “the conditions for deliberation,” that is, balanced and unbiased information was provided in both conditions in our experiment. Accordingly, we have added specific details describing these conditions to the relevant section (p. 4, l. 87-91).
- Page 6: States “….when participants deliberate on diverse perspectives of issues,…”
  - Diverse perspectives mitigate biases and adds strength to decision making
- Reply: We have explained in the relevant section how diverse perspectives can mitigate bias and strengthen decision-making (p. 7, l. 141-143).
- Page 8: States “…participants pursue mutually incompatible goals…”
  - Should add an example to clarify instances where goals may be incompatible
  - Sometimes more beneficial for participants to take opposing views, as controversy can often lead to additional funding and strengthen visibility vs a supportive view
- Reply: We have added specific examples of situations involving mutually incompatible goals. Additionally, we have included a discussion of the benefits of considering perspectives contrary to one’s original opinion (p. 8, l. 184 - p. 9, l. 187).
- Page 8: States “...participants work together to achieve shared objectives…”
  - Situations where there is a mutual benefit to participants provides motivation to work together to achieve shared objectives – add clarification
- Reply: We have provided specific examples of situations in which participants benefited from working together toward shared objectives (p. 9, l. 197-201).
- Research Aim and Hypotheses Section – Consider removing this section and modifying text on Page 12 where study hypotheses are shown to remove redundancy
- Reply: We have moved the “Research Aim and Hypotheses” subsection to your suggested location (p. 14, l. 302-317).
- Page 14 Experimental Overview: States “…asked to consider both positions regardless of their initial opinion.”
  - Possible that their own biases could influence their objectivity – source of uncertainty in study design
- Reply: We have discussed how various biases can influence participants’ initial opinions in the Discussion section (p. 34, l. 671-673; p. 36, l. 727-731).
- Table 1 and 3: Need to add more description and clarity to these tables – reader should be able to quickly view and understand results in a table without needing to delve too much into descriptive text before or after the table
- Reply: To improve clarity, we have revised the captions and column names of the tables (p. 17, l. 373-377; p. 18, l. 392).
- Table 4: Need to mention scale in header and link footnote (e.g., add * next to Table title and in front of foot note, place footnote directly under table). Footnote should also read “Informational materials are provided….”
- Reply: In the notes in Table 4, we have added the scales used in the post-questionnaire (p. 20, l. 419-422).
- Manipulation Section would have benefited from a table to summarize approach with a short description vs over two pages of text
- Reply: Based on your suggestion, we have summarized the contents of the manipulation section in a table to save space (p. 22, l. 461; Table 5).
- Page 33: States “…it is impossible to involve such stakeholders because the regions bearing the burden of the removed soil have not yet been determined…”
  - Study design could have included scenario where participants are asked to pretend soil will be placed in the regions where they live – consider adding as future recommendation in conclusions
- Reply: We agree that experiments using a scenario such as “if removed soil were to be managed in my own community” are necessary. However, because the Conclusions section is intentionally concise, we have added this point as a limitation regarding participant demographics (p. 37, l. 744- 748).
- Page 35: States “… cooperative controversy contexts can yield compromised solutions that reflect the perspectives of stakeholders on both sides…”
  - A solution oriented approach able to evaluate broader range of possible options and outcomes is not necessarily a compromise
- Reply: We have revised the phrasing in the relevant section to reflect that compromised solutions are not the only possible outcomes (p. 39, l. 785-789).

We have addressed all comments and suggestions to the best of our ability. Should there be any further points that require clarification or additional revision, please do not hesitate to let us know.

We hope that the revised manuscript now meets the requirements for publication in *PLOS One*. Thank you for your continued time and consideration.

Sincerely,

Yume Souma
